# Supplementation with live *Saccharomyces cerevisiae boulardii* during the initial 42 days of the feedlot phase in Nellore beef cattle

**DOI:** 10.1093/tas/txae097

**Published:** 2024-06-19

**Authors:** Lorena E de L M Bomfim, Kaique de S Nascimento, Alana M de M Calaça, Luan de O M Silva, Emmanuel Arnhold, Victor R M Couto, Yasmin M Barreto, Lucas J Mari, Mateus C Santos, Gauthier Marine, Eric Chevaux, Juliano J de R Fernandes

**Affiliations:** Departamento de Zootecnia, Universidade Federal de Goiás, Goiânia, GO, Brazil; Departamento de Zootecnia, Universidade Federal de Goiás, Goiânia, GO, Brazil; Departamento de Zootecnia, Universidade Federal de Goiás, Goiânia, GO, Brazil; Departamento de Zootecnia, Universidade Federal de Goiás, Goiânia, GO, Brazil; Departamento de Zootecnia, Universidade Federal de Goiás, Goiânia, GO, Brazil; Departamento de Zootecnia, Universidade Federal de Goiás, Goiânia, GO, Brazil; Departamento de Zootecnia, Universidade Federal de Goiás, Goiânia, GO, Brazil; Lallemand Brasil Ltda., Aparecida de Goiânia, Brazil; Lallemand Brasil Ltda., Aparecida de Goiânia, Brazil; Lallemand SAS, Blagnac, France; Lallemand SAS, Blagnac, France; Departamento de Zootecnia, Universidade Federal de Goiás, Goiânia, GO, Brazil

**Keywords:** carcass yield, feed efficiency, feedlot, gain, live yeast, probiotic

## Abstract

This study aimed to assess the effect of *Saccharomyces cerevisiae boulardii* CNCM I-1079 supplementation during the initial feeding period on the performance of Nellore bulls in a feedlot system. One hundred ninety-eight Nellore bulls were used in a completely randomized block design, with blocking based on weight within each treatment group: light (331.4 kg; 4 pens), medium (349.7 kg; 4 pens), and heavy (362.5 kg; 3 pens). The treatments included CON—a basal diet, and SCB—basal diet plus a probiotic (*Saccharomyces cerevisiae boulardii* CNCM I-1079; 1.0 × 10^10^ CFU/head/d). Experimental diets were administered for the first 42 d (21 d in the step-up phase and 21 d in the finishing diet −870 g concentrate/kg dry matter [**DM**]). Subsequently, both treatment groups were transitioned to the same basal diet for an additional 76 d, completing 118 d on feed. Linear regression analysis was conducted for dry matter intake (**DMI**) data. During the initial 42 d, DMI tended to be higher for SCB (*P* = 0.09); also bulls fed SCB reached the plateau of the curve at 9.17 kg DMI/d earlier (39 d, R^2^ = 0.97) than those fed CON (43 d; R^2^ = 0.96) diets. For the first 42 d, the SCB treatment exhibited higher final weight (393.0 vs. 401.4 kg, *P* = 0.02), total gain (49.3 vs. 53.5 kg, *P* = 0.02), daily weight gain (1.124 vs. 1.274 kg, *P* = 0.02), and G:F (0.174 vs. 0.188, *P* = 0.04). Over the entire 118-d period, SCB-fed bulls had greater final body weight (509.5 vs. 518.0 kg, *P* = 0.02), total body weight gain (163.7 vs. 170.3 kg, *P* = 0.01), and average daily gain (1.366 vs. 1.420 kg, *P* = 0.01). The feed efficiency of SCB-supplemented bulls was 8.05% higher than CON (*P* = 0.04), and the final carcass weight was 1.69% greater for animals fed SCB (283.8 vs. 288.6 kg, *P* = 0.04). Total carcass weight gain (110.9 vs. 114.7 kg) and daily carcass weight gain (0.924 vs. 0.956 kg) tended (*P* = 0.06) to increase by 3.46% in SCB-fed animals compared with those fed CON. Gain yield, carcass conversion, and carcass yield did not differ between treatments. There were no significant differences in the apparent digestibility of DM, crude protein, neutral detergent fiber, and ether extract between treatments. However, starch digestibility (92.7% vs. 88%) was greater for the control treatment (*P* < 0.001). Including live *Saccharomyces cerevisiae boulardii* yeast as a probiotic supplement during the initial 42 d in the feedlot enhanced early-stage growth performance in Nellore bulls. Notably, this supplementation carried over carcass gain over the entire feedlot period.

## Teaser

Supplementation with the probiotic *Saccharomyces cerevisiae boulardii* (SCB) positively impacted the performance of Nellore cattle in the first 42 d of feeding, resulting in optimal adaptation. The excellent performance of the animals in the first 42 d was maintained until the final period of the feedlot, during which the supplemented animals (SCB) showed better performance and carcass characteristics for a total of 118 d.

## INTRODUCTION

Cattle adaptation to feedlot finishing diets is crucial to mitigate the risks of subacute and acute rumen and hindgut acidosis. This is the period where forage is replaced with highly fermentable carbohydrates and the animals transition from grazing behavior to closed pens leading to a shift in the ruminal microbiota toward starch-fermenting bacteria dominance (Nocek and Taminga, 1991), highlighting the imperative of proficient nutritional strategies for enhancing performance via the adept modulation of ruminal microbiota adaptation (Ricci et al., 2022).

Probiotic additives have the potential to modulate the microbiota, enhance gastrointestinal health, and fortify the immune system during the receiving period (Siverson et al., 2012). The yeast *Saccharomyces cerevisiae* (SC) stands out as one of the extensively researched probiotics in ruminant nutrition. Among the multifaceted contributions, a crucial role in fiber digestibility stands out prominently (Nagaraja, 2012), the maintenance of ruminal pH (Helal and Abdel-Rahman, 2010), and the facilitation of the adaptation of ruminal microorganisms to high-grain diets. In contrast, the yeast strain *S. cerevisiae sub. boulardii* (SCB), extensively examined in nonruminants (Vohra et al., 2016) and humans (Pais et al., 2020), serves as an enhancer of intestinal health, actively suppressing pathogens within the intestinal tract. However, research on the application of SCB for ruminants is more limited.

Utilized as a direct-fed microbial, SCB can survive in the rumen, either alone or in combination with other microorganisms (Puniya et al., 2015). Adding SCB to the starter feed has been shown to stimulate the total population of Lactobacilli bacteria at weaning, potentially influencing immune system modulation (Fomenky et al., 2017). The inclusion of SCB in the starter feed has demonstrated reduced preweaning mortality rates in dairy calves (Magalhães et al., 2008), lower incidence of diarrhea in heat-stressed dairy calves (Lee et al., 2019) and in veal calves (Villot et al., 2019) fed yeast in the milk replacer, and modulation of the immune system in newborn calves fed SCB in the milk replacer for the first seven days of life (Villot et al., 2020).

In beef cattle, however, recent results have reported either improvement in performance or in the health status of animals. High-risk heifers receiving SCB for the first 45 d in a feedlot, exhibited improved performance, with enhanced carcass quality and decreased liver abscesses (Theurer et al., 2019). Conversely, although no performance differences were depicted, Keyser et al. (2007) observed a reduction in respiratory morbidity in newly received heifers fed 10 billion CFU/d of SCB, as another key achievement for beef production.

Given the limited information along with a growing demand for natural alternatives to enhance growth performance and feed efficiency in beef cattle, we hypothesized that probiotic-fed cattle during the initial 42 d of the feedlot phase would result in additional benefits to cattle performance and efficiency. The objective of this study was to investigate the effects of SCB supplementation on the performance, carcass traits, and apparent digestibility of nutrients in Nellore bulls during the initial feeding period.

## MATERIAL AND METHODS

All procedures were approved by the Ethics and Animal Welfare Committee of the Federal University of Goiás, Goiânia - GO, Brazil (approval no. 124-19).

### Animals, Diets, Sample Collection, and Data Collection

One hundred ninety-eight Nellore bulls, aged between 20 and 24 months, with an initial body weight of 346.6 ± 14.4 kg, were selected for the study. All the animals were raised in a grazing system on the same farm. The weighing was conducted at the farm of origin, and subsequent blocking by weight was implemented within each treatment group: light (331.4 kg; 4 pens), medium (349.7 kg; 4 pens), and heavy (362.5 kg; 3 pens), with each pen accommodating nine animals. During the 22-h transportation to the experimental site, feed and water were withheld from the animals. The feedlot was located at the Federal University of Goias. Upon arrival, the animals underwent another weighing, considering the experiment’s initial weight.

Within each block, pens were randomly assigned to one of two treatments (11 pens/treatment): Control (CON)—basal diet, and SCB—basal diet plus probiotic supplementation at 0.5 g/d per animal with live yeast *Saccharomyces cerevisiae boulardii* CNCM I-1079 (ProTernative 20; Lallemand Animal Nutrition, dose: 1.0 × 10^10^ CFU/head/d) for 42 d, spanning 21 d in the adaptation period and 21 d in the final diet (Table 1). Animals (9 per pen) were allocated in soil-surfaced outdoor pens (77 m^2^) with a 7-m feedbunk and a concrete water trough.

**Table 1.** Ingredient and chemical composition of experimental diets during 118 d.

Ingredient, g/kg DM	Experimental diet^1^			
	ADAP 1	ADAP 2	ADAP 3	FINAL
Sugarcane bagasse	300	230	140	130
Ground corn	523	611	728	747
Soybean meal	160	140	110	93
Urea	4.4	5.1	6.1	8.1
Mineral mix^2^	11.7	13.7	16.3	21.7
Chemical composition^3^ (g/kg DM)				
DM (g/kg as fed)	729.7	723.2	721.5	722.2
Crude protein	142	131	128	120.3
Ether extract	14.3	16.5	22.1	25.7
aNDFom	349.3	288	270.5	276.9
Ash	53.6	52	48.2	42
Organic matter	948.8	950.2	957.2	958.6
NEm, Mcal/kg	1.79	1.82	1.85	1.86
NEg, Mcal/kg	1.15	1.18	1.21	1.22
Starch	389.6	450.3	519	524

^1^Adap: composition of the step-up diets during the adaptation phase. Final: Finishing diet. Treatments: Control (with 28 ppm of monensin); and SCB—control with monensin plus probiotic (1.0 × 10^10^ CFU/head/d of *Saccharomyces cerevisiae boulardii* CNCM I-1079).

^2^Calcium: 240 g/kg; phosphorus: 12.5 g/kg; sodium: 100 g/kg; magnesium: 30 g/kg; copper: 500 mg/kg; manganese: 1,000 mg/kg; zinc: 1,500 mg/kg; cobalt: 7.5 mg/kg; iodine: 25 mg/kg; selenium: 12.5 mg/kg; monensin 28 mg/kg.

^3^DM, dry matter; aNDFom, neutral detergent fiber with heat-stable amylase and exclusive of residual ash. NEm, net energy for maintenance; NEg, net energy for gain. Estimated from feed composition (NASEM, 2016).

Previous studies have indicated that within 42 d, ProTernative contributes to intestinal health during stressful periods, such as adaptation in the feedlot (21 d), characterized by a significant passage of concentrate through the gastrointestinal tract (Theurer et al., 2019; Smith et al., 2020). The experimental period was divided into 21 d of adaptation to the finishing diet and 97 d on the finishing diet with 87% concentrate.

After the 42-d experimental period, the additive was removed from the SCB treatment, and the animals were weighed without fasting, deducting 4% of the final live weight (Valadares Filho et al., 2016) to prevent interference with the dry matter intake (**DMI**) of the animals.

To maintain the dry matter (**DM**) content of the total mixed ratio (**TMR**) at 72%, water was added as needed, following the formula ([DMI kg/72%] − [NMI kg] × 100), where DMI is the average dry matter intake of the animals observed on the day, and NMI is the sum of the intake per animal of all ingredients in the diet on an as-fed basis (Table 1). During the adaptation period, the inclusion of sugarcane bagasse was gradually reduced in the DM of the diet, utilizing the step-up protocol with step 1 (7 d), step 2 (7 d), step 3 (7 d), and the final diet (97 d) incorporating 30%, 23%, 14%, and 13% of sugarcane bagasse, respectively. The concentrate was comprised of corn, soybean meal, urea, and a mineral mix. The SCB product (0.5 g/animal) was calculated based on the number of animals in the treatment and incorporated into the mineral mix. Therefore, two premixes were prepared, one with SCB and one without the live yeast. Concentrate, sugarcane bagasse, and mineral premix were blended daily as a TMR in a mixer wagon.

Feed was provided ad libitum once a day at 1330 h. The feed bunk score, determined at 1300 h, allowed for approximately 3% orts, with adjustments made to increase or decrease it by 0.400 kg/DM (bunker empty) for more than 4% (bunker full) and 0.250 kg/DM (bunker between 1% ≤ orts < 4%) per animal in the adaptation and final diets, respectively. The DMI was calculated as the difference between feed offered and orts. Feed and orts were collected weekly and stored at −10°C for later chemical analysis.

For apparent digestibility analyses, fecal samples were collected after spontaneous defecation for three consecutive days and at two different shifts (0600 h/1800 h) before additive withdrawal (days 39 to 41). Fecal samples were obtained from at least eight animals per pen (Del Bianco et al., 2016). Immediately following the spontaneous defecation of the animals, at the times mentioned above, small amounts of the top part of the feces were collected, and the pool was prepared. Samples were pooled by pen and by day, then stored at −20°C for subsequent analysis.

### Carcass Traits

On the last day of the experiment (118 d), the animals underwent weighing after a 12-h fasting from solids to determine the final weight. Subsequently, they were transported to the Vale do Cedro slaughterhouse, a commercial plant situated in the municipality of Ihumas—GO, Brazil, located 55 km away from the experimental feedlot.

Following slaughter, carcass weights were recorded, considering a 50% initial carcass yield (Ito et al., 2012), given the absence of a reference slaughter at the beginning of the experiment. Yield gain was calculated using the formula: (ADG carcass /ADG live weight), where ADG live weight represents the average daily gain, and ADG carcass is the average daily gain in carcass, expressed as ([FCW − ICW]/Experiment days), with FCW denoting final carcass weight and ICW representing initial carcass weight. Carcass conversion data were obtained using the equation: CC = ([15/ADG carcass] × DMI), where 15 is a 15-kg unit of carcass.

### Chemical Analysis and Net Energy Estimates

Feed, orts, and fecal samples were dried in a forced-air circulation oven (Tecnal, Piracicaba, Brazil) at 55 °C for 72 h and ground to pass a 1-mm sieve in a Wiley-type mill (Marconi, Piracicaba, Brazil). Chemical analyses were performed following AOAC (2023) guidelines: DM (method 930.15); ash (method 942.05); crude protein (**CP**, method 976.05); ether extract (**EE**, method 920.39), and starch (method 996.11). Additionally, neutral detergent fiber (aFDNom) was determined according to Mertens (2002), utilizing thermostable amylase (Tecnoglobo, Curitiba, Brazil) without sodium sulfite and exclusive of residual ash.

Daily energy gain (EG; Mcal/d) was estimated by the equation: EG = (0.0557 × BW^0.75^) × ADG^1.097^, where BW is the average body weight, and ADG is the average daily gain (kg; NASEM, 2016). Metabolizable energy (ME; Mcal/d) was estimated as: ME = 0.077 × BW^0.75^ (Lofgreen and Garret, 1968). Net energy for maintenance (NEm) and gain (NEg) were calculated as follows (Zinn and Shen, 1998): NEm = (−*b*−((*b*²)−(4*ac*))^0.5^))/2, where *a* = −0.41 × ME, *b* = 0.877 × ME + 0.41 × DMI + EG, and *c* = −0.877 × DMI; NEg = 0.877 × DMI.

For apparent digestibility calculation, indigestible neutral detergent fiber (**iNDF**) served as an internal marker (Cole et al., 2011) to estimate fecal excretion. Samples of the diets supplied, orts, and feces were ground to pass a 2-mm sieve in a Wiley-type mill and analyzed for indigestible NDF after 288 h of in situ rumen incubation (Huhtanen et al., 1994). Fecal excretion was calculated as FE (g/d) = iNDF intake (g/d)/fecal iNDF (%).

Nutrients estimated for apparent digestibility were DM, aNDFom, CP, EE, and starch. Apparent digestibility coefficients were calculated as follows: (Nutrient in TMR (g/kg DM) × TMR intake (kg DM/d) − (Nutrient in feces (g/kg DM) × FE (kg DM/d)/(Nutrient in TMR (g/kg DM) × TMR intake (kg DM/d)) × 100, where TMR intake is the average intake of the animals in the digestibility trial period.

### Statistical Analysis

Performance, carcass traits, and digestibility data were analyzed using a completely randomized block design, applying the PROC MIXED procedure in SAS (2000) - (version 9.4; SAS Institute, Cary, NC, USA). The pen was considered the experimental unit for all variables. The statistical model is as follows:


$$\begin{array}{l} Yij=\propto +ti+bj+Eij \end{array}$$

In this equation, α: constant; *ti*: treatment effect; *bj*: block effect; and *Eij*: error.

A linear regression analysis was performed to determine the time to reach stabilized intake, utilizing the mean DMI data between treatments over the 118 d of the experimental study. It was obtained by R software, using the easyreg package (Arnhold, 2018). The variables were compared using the *F*-test at a 5% significance level. A significant difference (*P* < 0.05) and trend were considered for all variables when 0.05 ≤ *P* < 0.10. Experimental unit outliers were defined as such when their standard deviation deviated above or below 2.5% of the corresponding pen mean. Hence, experimental units 8 and 22 of the control treatment were considered outliers, considering performance variables such as final weight, daily weight gain, and total weight gain.

## RESULTS

**Figure 1.**
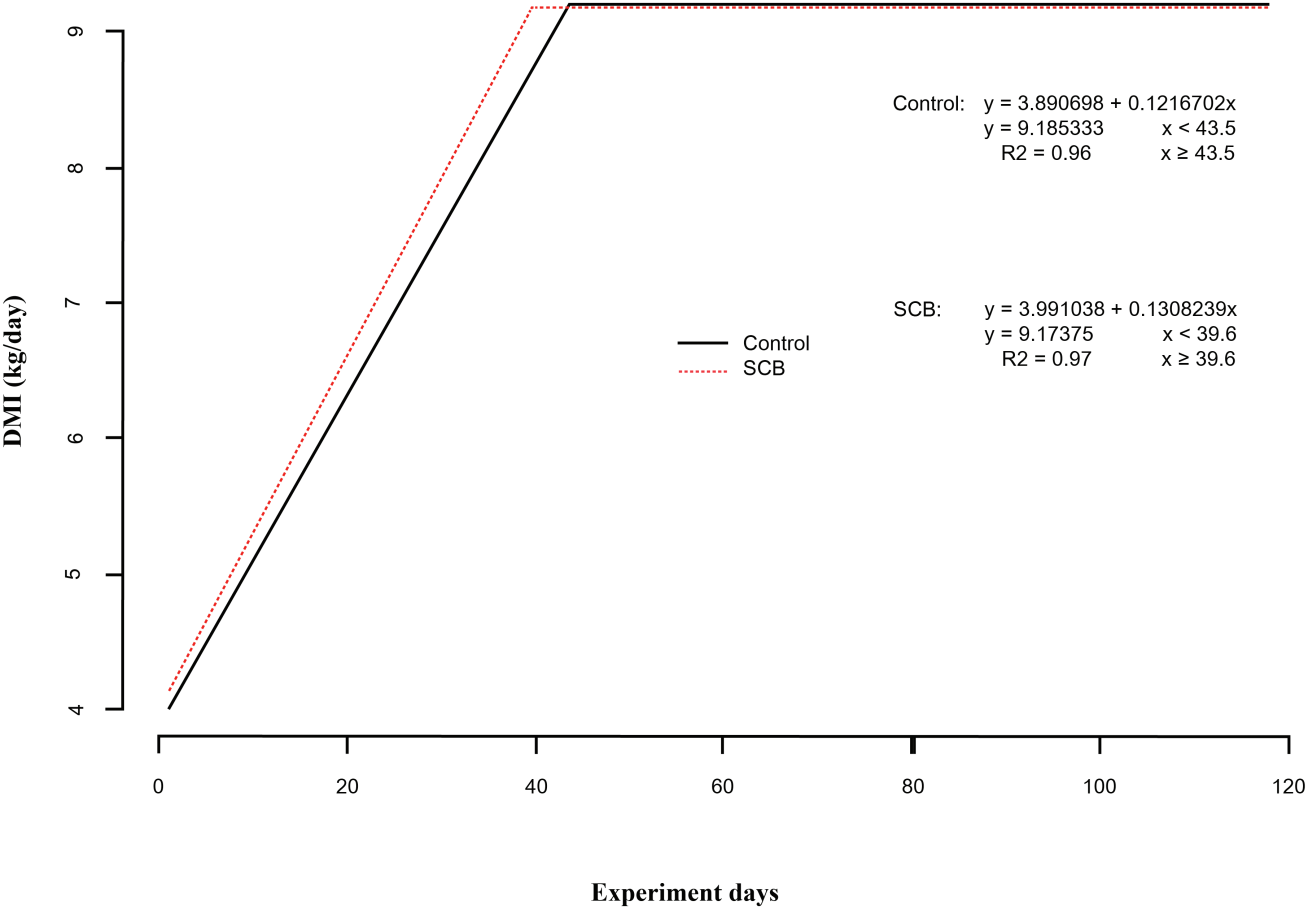
Control: DMI plateau of control treatment animals (R^2^ = 0.96; 9.18 kg/DM). SCB: DMI plateau of SCB treatment animals (R^2^ = 0.97; 9.18 kg/DM).

During the initial 42 d, DMI tended to be greater for SCB inclusion (*P* = 0.09; Table 2). However, bulls fed SCB plateaued their curve earlier at 9.17 kg DMI/d (39 d, R^2^ = 0.97) than those fed CON (43 d; R^2^ = 0.96) diets (Fig. 1). During this phase, final weight (*P* = 0.02), total body weight gain (*P* = 0.02), and average daily gain (*P* = 0.02) were greater for the SCB group compared with CON. The feed efficiency of SCB-supplemented bulls was 8.05% greater than that of the animals receiving CON (*P* = 0.04). There were no significant differences in net energy for gain (NEg), net energy for maintenance (NEm), or the ratio between observed and expected NEm and NEg (*P* = 0.17; Table 2).

**Table 2.** Performance data during supplementation in the first 42 d

Variable	Control	SCB	SEM	*P*-value
DMI, kg/d	6.4	6.7	0.18	0.09
Initial BW, kg^1^	345.8	347.7	2.57	0.46
Final BW, kg^2^	393.0	401.4	3.32	0.02
Total gain, kg	49.3	53.5	2.49	0.02
ADG, kg	1.124	1.274	0.0598	0.02
G:F	0.174	0.188	0.0062	0.04
Energy				
NEm, Mcal/kg	1.59	1.64	0.038	0.17
NEg, Mcal/kg	0.98	1.03	0.033	0.17
Observed:expected NEm	0.85	0.88	0.138	0.17
Observed:expected NEg	0.81	0.84	0.018	0.17

^1^Initial BW on arrival to the feedlot unity.

^2^Final BW without fasting, discounting 4% of the BW.

Abbreviations: NEm, net energy for maintenance; NEg, net energy for gain.

Considering the 118 d of the experiment, the final body weight (*P* = 0.02), total body weight gain (*P* = 0.01), and average daily gain (*P* = 0.01) were greater for SCB than CON. However, there were no significant differences in NEg, NEm, or the ratios between observed and expected NEm and NEg (*P* = 0.17; Table 3).

**Table 3.** Performance and carcass trait data during SCB supplementation for 118 d

Variable	Treatment		SEM	*P*-value
	Control	SCB		
Final BW, kg^1^	509.5	518.0	3.57	0.02
Total BW gain, kg	163.7	170.3	2.46	0.01
ADG, kg	1.366	1.420	0.0200	0.01
DMI, kg/d	8.16	8.34	0.106	0.12
DMI, % BW	1.91	1.92	0.024	0.51
G:F	0.167	0.170	0.003	0.23
Energy				
NEm, Mcal/kg	2.06	2.08	0.024	0.35
NEg, Mcal/kg	1.40	1.42	0.021	0.35
Observed:expected NEm	1.11	1.12	0.008	0.35
Observed:expected NEg	1.14	1.16	0.012	0.35
Carcass traits				
Final carcass weight, kg	283.8	288.6	2.26	0.04
Carcass dressing, %^2^	55.8	55.7	0.03	0.71
Carcass daily gain, kg	0.924	0.956	0.0161	0.06
Total gain of carcass, kg	110.9	114.7	1.91	0.06
Gain yield, %	67.9	67.3	0.007	0.39
Carcass conversion	132.5	130.7	1.81	0.35

^1^Final BW after 12 h of fasting.

^2^The inicial carcass dressing considered was 50%.

Abbreviations: NEm, net energy for maintenance; NEg, net energy for gain.

Carcass weight was 1.69% greater for SCB (*P* = 0.04). Total carcass weight gain and daily carcass weight gain (0.924 vs. 0.956 kg) tended (*P* = 0.06) to increase for SCB compared with CON. Gain yield (*P* = 0.39), carcass conversion (*P* = 0.35), and carcass dressing (*P* = 0.71) were similar between treatments (Table 3).

There were no significant differences in the apparent digestibility of DM, CP, NDF, and EE. However, starch digestibility was 4.7% greater in the animals fed CON compared with those in the SCB group (*P* < 0.001; Table 4).

**Table 4.** Apparent digestibility of nutrients in animals fed control and SCB treatments during the 42-d period

Variable	Treatment		SEM	*P*-value
	Control	SCB		
Apparent digestibility, %				
Dry matter	63.7	63.5	1.42	0.91
Crude protein	60.1	58.0	1.73	0.42
Neutral detergent fiber	32.7	29.9	3.16	0.51
Ether extract	50.0	53.5	4.03	0.57
Starch	92.7	88.0	0.32	<0.001

## DISCUSSION

During the initial 42 d of the experimental period, animals supplemented with SCB demonstrated superior performance compared to the CON group.Similar findings were reported by Theurer et al. (2019) for high-risk heifers supplemented with SCB for 45 d, resulting in decreased bovine respiratory disease first treatment. This supplementation also led to improved carcass quality and reduced liver abscesses (Theurer et al., 2019). Low-risk steers, when fed live yeast for the first 47 d on feed, exhibited improved weight gain and greater ADG, although no cumulative effects were observed (Smith et al., 2020).

The observed positive effects of SCB on gastrointestinal tract health and immune system function suggest its potential to enhance overall health and performance (Theurer et al., 2019). This study represents the first exploration of the potential use of SCB in adult animals to improve digestibility and performance in a feedlot system.

In the period of adaptation to the diet, the growth rate of amylolytic bacteria increases exponentially with the increase in nonfibrous carbohydrates potentially leading to low rumen pH due to elevated organic acids and lactate accumulation. This condition can manifest as subacute ruminal acidosis (**SARA**), characterized by intermittent periods of low ruminal pH between acute and chronic durations (Oetzel, 2017). SARA adversely affects DMI and feed efficiency, with chronic cases associated with the development of rumenitis, liver abscesses, and hindgut acidosis. Furthermore, in situations where acidosis occurs in the intestine, it may contribute to the development of leaky gut syndrome, characterized by increased intestinal permeability (Rodriguez-Jimenez et al., 2019; Fu et al., 2022). This syndrome arises from factors such as inflammation, which damages the gut wall, allowing the translocation of lipopolysaccharide (**LPS**) within the gut ( Khiaosa-ard and Zebeli, 2018). One hypothesis places LPS as a possible primary factor responsible for peripheral inflammation in the intestine and in the rumen (Guo et al., 2017; Khiaosa-Ard and Zebeli, 2019; Fu et al., 2022). Recent studies have observed a correlation between animals receiving a diet high in fermentable carbohydrates, increased inflammatory processes (Zhang et al., 2016), and higher levels of LPS in the rumen (Zhang et al., 2019).

The probiotic mechanism of SCB, reviewed in humans (Im and Pothoulakis, 2010; Pais et al., 2020), includes inhibiting pathogenic bacterial products, trophic effects on the intestinal mucosa, and modifying host signaling pathways involved in inflammatory processes and noninflammatory bowel diseases. These effects highlight the intestine as the primary target of SCB action, a phenomenon demonstrated across various mammal species, including humans and ruminants.

In ruminants receiving high-grain diets, a portion of the starch may evade rumen degradation, finding its way to digestion in the small intestine or fermentation in the large intestine. Hindgut acidosis manifests as heightened hindgut fermentation, leading to an excess of volatile fatty acids, decreased luminal and fecal pH, and damage to the intestinal epithelium. Gressley et al. (2016) noted that animals fed SCB exhibited an enhanced fecal score and a tendency to reduce fecal short-chain fatty acids after an oligofructose challenge. In the current study, starch digestibility for the SCB treatment was 5.4% lower (Table 4) without compromising performance. These findings collectively suggest that SCB stabilized the intestinal environment by reducing starch fermentation, potentially leading to improved intestinal health and consequently, enhanced overall performance. In addition, SCB supplementation has been shown to reduce severe liver abscesses, possibly by reducing the adherence and translocation of Gram-negative bacteria across the intestinal wall (Theurer et al., 2019), acting on mitigating the inflammatory potential of circulating LPS.

Contrary to some studies showing a decrease in the ruminal population of *Prevotella* sp. following probiotic live yeast addition to a high-grain diet fed to steers (Ogunade et al., 2020), SCB in this study did not compromise performance despite a lower starch digestibility. A blend of *Meghasphaera elsdenii* and *S. cerevisiae*, assessed at increasing levels in a continuous dual-flow culture system simulating high-starch diets, resulted in an 11.9% reduction in starch digestibility (Monteiro et al., 2022). Additionally, *S. cerevisiae* demonstrated competition with other starch-utilizing bacteria, effectively fermenting starch (Lynch and Martin, 2002) and thereby preventing lactate accumulation in the rumen.

Based on the above, we suggest that the positive outcomes observed at the conclusion of the experiment and in the carcass characteristics of SCB-supplemented animals stem from a well-adapted microbiota and a functional epithelium in the rumen and intestinal tract. These factors worked together to produce cumulative effects resulting in these benefits. Cattle with poor adaptation exhibited higher rumenitis scores and lower ADG. Moreover, the rumen epithelium reaches a developmental plateau, with the absorptive surface area of the rumen wall being the morphometric variable most correlated with the speed of short-chain fatty acid absorption (Estevam et al., 2020). Comparing the probiotics *S. cerevisiae* and SCB, it is evident that both can positively impact animal performance and fermentation (Piños-Rodríguez et al., 2008; Ogunade et al., 2020). SCB also demonstrates modulation of animal health (He et al., 2017; Lee et al., 2019; Villot et al., 2019) and the immune system (Kayser et al., 2019), all mechanisms leading to energy and nutrient sparing by the host that are turned in protein accretion and growth.

Based on this review, we can assume that enhanced gastrointestinal tract health in SCB-fed animals in the present study, improved the passage rate of the feed, demonstrated by the lower starch digestibility and led to a higher DMI and faster intake rate during the experiment (Fig. 1). The improved growth performance and carcass characteristics were likely attributable due to increased intake of nutrients without triggering any intestinal fermentation excess and inflammation.

## CONCLUSION

Probiotic supplementation with SCB during the first 42 d in the feedlot system improved early growth performance, with a notable carry-over effect on carcass gain throughout the entire feedlot period. The reduced starch digestibility for the SCB treatment may contribute to the overall better performance of supplemented animals through a more stable intestinal metabolism. This study also highlights the necessity for further research to elucidate how SCB modulates the microbiota.
